# Clinical Use of Inhibitors of HIV-1 Integration: Problems and Prospects

**Published:** 2011

**Authors:** S.P. Korolev, Yu.Yu. Agapkina, M.B. Gottikh

**Affiliations:** Department of Chemistry, Lomonosov Moscow State University; Belozersky Research Institute of Physico-Chemical Biology, Lomonosov Moscow State University

**Keywords:** HIV-1 integrase, inhibition, mechanism of action, raltegravir

## Abstract

The HIV-1 integrase enzyme is responsible for one of the key stages of retroviral replication; it acts as a catalyst for the integration of viral cDNA into the cell’s genome. Inhibitors of HIV-1 integration have been under development for over 10 years; yet, only one integration inhibitor, raltegravir, has been approved for clinical use so far. Raltegravir binds two metal ions in the enzyme’s active centre and blocks one of the integration stages: the strand transfer. Unfortunately, the clinical use of raltegravir results in the development of viral resistance among some patients. Several more HIV-1 integration inhibitors are undergoing clinical trials at the moment. However, the structure and mechanism of action of those are similar to raltegravir, which results in the emergence of cross resistance with raltegravir. The present review is focused on the history of the development and clinical trials of raltegravir and its analogues, the problems connected with the emergence of viral resistance to integration inhibitors, and the prospect of their future clinical use.

## INTRODUCTION

The acquired immunodeficiency syndrome (AIDS) began as one of the most dramatic epidemics of the late 20 ^th^ – early 21st centuries. AIDS is caused by the human immunodeficiency virus (HIV) afflicting the immune system of the organism. Ukraine and Russia have some of the highest rates of the spread of the HIV infection in the world. HIV prevalence among the adult population in Russia is over 1.1% [1], according to some estimates. This is precisely why the development of effective therapeutic drugs to control the spread of the virus is particularly urgent for Russia.

HIV afflicts primarily the cells of the immune system: the CD4 ^+^ Т-lymphocytes, macrophages, and dendritic cells. The stock of CD4 ^+^ cells is gradually depleted, resulting in the subsiding of cell immunity. When a critical lymphocyte level is achieved, the organism becomes easy prey for opportunistic infections [2]. The following stages of the HIV-infection can be distinguished in the absence of antiretroviral therapy: the primary infection – acute HIV syndrome that ends with the extinction of clinical symptoms and seroconversion; the latent stage (symptom-free chronic HIV infection); symptomatic HIV infection (AIDS), which is often accompanied by the development of opportunistic infections; and the terminal stage (death) [2].

**Fig. 1:**
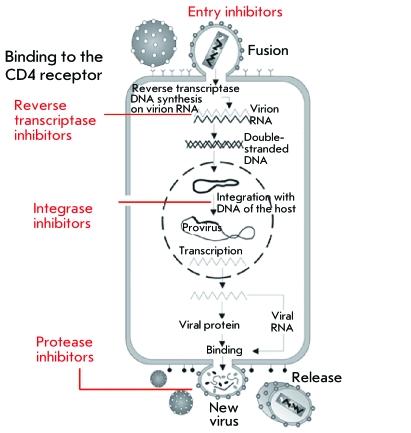
HIV-1 replication cycle and HAART targets.

The replication cycle of HIV-1 can be tentatively divided into two phases: the early phase and the late phase ( *Fig. 1* ) [2, 3]. At the early stage of the life cycle, viral particles specifically bind onto the CD4 surface protein thanks to the specific interaction between the viral coat glycoprotein gp120 and the N-terminal domain of the immunoglobulin of the CD4 protein. The binding onto the CD4 receptor allows gp120 to bind to the coreceptors (CCR5 or CXCR4) on the surface of a target cell, as well. After the binding of g120 to coreceptors, glycoprotein gp41 is incorporated into the cell membrane, resulting in the fusion of the viral coat and the cell membrane yielding a pore, through which the viral core penetrates into the cell cytoplasm [2]. After the fusion, the virus sheds its coat, and the process of reverse transcription begins. The reverse transcription of genomic RNA is carried out via the viral enzyme; reverse transcriptase, in cytoplasm. The product of reverse transcription, double-stranded cDNA, is transported into the nucleus within the pre-integration complex, which comprises a number of viral proteins, such as integrase (IN), the matrix protein (MA), reverse transcriptase, the nucleocapsid protein (NC), and the regulatory protein Vpr (Viral Protein R) [4, 5], as well as the cell proteins Ku [6], HMG I(Y) [7], BAF [8], and LEDGF/p75 [9]. The nuclear localization of IN, MA, Vpr [5], and LEDGF/p75 [9] is ensured by nuclear localization signals. After it is transported into the nucleus, a DNA copy is integrated, i.e., covalently incorporated into the genome of the host cell due to the catalytic activity of IN [3]. The late phase of the replication cycle of HIV-1 begins with the regulated expression of the proviral genome; then, processing of the synthesized viral proteins with viral protease occurs, followed by the assembly of new virions, which are released from the cell and infect new target cells, ultimately terminating the life cycle of the virus [2, 3].

Highly active antiretroviral therapy (HAART), which at the time of writing comprises 25 drugs, is used in the treatment of HIV infection [10]. These drugs mostly include nucleoside and non-nucleoside inhibitors of reverse transcriptase of HIV-1 and protease inhibitors. Moreover, entry inhibitors have recently been designed: maraviroc, which blocks the interaction between gp120 and CCR5, and enfuvirtide, which interacts with gp41 glycoprotein ( *Fig. 1* ). At the end of 2007, the U.S. Food and Drug Administration (FDA) approved the first integration inhibitor, Isentress ^TM ^ drug, also known as raltegravir (MK-0518), an anti-AIDS agent [11].

The stage at which the viral DNA is integrated into cell DNA is one of the key stages in the replication cycle of HIV-1; therefore, IN catalyzing is considered to be one of the most attractive targets for HIV-1 inhibitors. It has been demonstrated that а virus containing a defective IN, which is incapable of catalyzing the integration of viral DNA, cannot be reproduced in cell culture [12]. Moreover, IN does not have a cell equivalent; therefore, the inhibitors that specifically suppress its catalytic activity are supposed to have no effect on the cell processes and should be less toxic for the cell and the entire organism in comparison to the inhibitors of other stages of the HIV replication cycle. Over many years, the development of integration inhibitors has been pursued, with various drugs capable of blocking IN described in minute detail in numerous reviews [13–19]. The present review is devoted to state-of-the-art studies in the field of application of raltegravir and its analogues as HAART components.

## INTEGRASE STRUCTURE AND INTEGRATION MECHANISM

The integration process begins in cytoplasm and comprises several stages [20–22]. A DNA copy of the viral RNA contains long terminal repeats at both ends, which consist of three fragments: U3, R, and U5. At a distance of two nucleotides from the 3’ terminus of each DNA strand, there is a conservative CA dinucleotide, which is found in the long terminal repeats of all retroviruses. Within the preintegration complex, IN recognizes the nucleotide sequences located at the termini of regions U3 and U5 of the viral cDNA, binds to them, and catalyzes the reaction of 3’-terminal processing. This reaction represents the endonuclease cleavage of the viral cDNA, resulting in the removal of the GT dinucleotide from the 3’ terminus in each strand. Substrate cleavage is caused by the nucleophilic attack on the phosphate group between the second and third nucleotides by a water molecule [20–22].

**Fig. 2:**
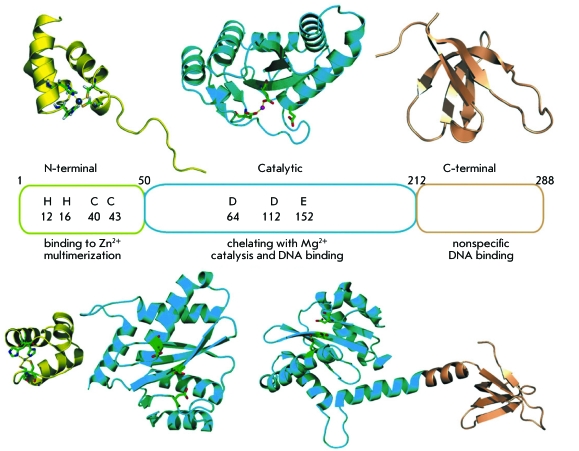
Structural domains of HIV-1 integrase [14].

The pre-integration complex is then transported into the nucleus, where IN catalyzes the strand transfer (ST) stage. This stage is represented by the re-esterification reaction, which involves the nucleophilic attack on the internucleotide phosphates of both strands of cellular DNA (DNA targets) by 3’-hydroxylic groups of the processed strands of viral (substrate) DNA, yielding a covalent product. The internucleotide bonds located in different strands of the DNA target at a distance of 5 np from each other undergo re-esterification. The completion of integration requires the following processes: processing of the 5’-termini of viral DNA, polymerase addition of five lacking nucleotides, and ligation, which are performed with the participation of cell proteins [20].

In the integration process, IN is required to bind two termini of the viral DNA to the cell DNA. However, the data on the interaction between IN and DNA as yet remains quite limited. HIV-1 integrase is a protein consisting of 288 amino acid residues (32 kDa) encoded by the *pol* viral gene. IN is translated within the Gag-Pol polypeptide, which is subsequently cleaved into separate proteins by a viral protease [21]. It has been demonstrated by partial proteolysis and targeted mutagenesis that three domains can be isolated in the enzyme structure: the N-terminal domain, comprising the amino acid residues 1–50; the catalytic domain formed by residues 51–212; and the C-terminal formed by the amino acid residues 213–288 ( *Fig. 2* ) [20].

The N-terminal domain contains two histidine and two cysteine residues, which are conservative in retroviral integrases and retrotransportases [20]. These residues coordinate the zinc ion and participate in the formation of a catalytically active IN multimer, since it has been demonstrated that the coordination of Zn ^2+ ^ ions stimulates IN multimerization and its activity [23]. The catalytic domain of IN contains the triad of invariant amino acid residues (D64, D116, and E152 in HIV-1) which form the active centre of retroviral integrases. The catalytic domain participates in the binding of the termini of viral DNA due to their interaction with conservative amino acid residues of the domain (primarily Q148, K156 and K159). For the integration to be possible, IN demands the presence of ions of cofactor metal ions (Mg ^2+^ or Mn ^2+^ ), which are coordinated with two residues from the catalytic triad (D64 and D116) [21]. The least conservative C-terminal domain forms the Src homology 3-like fold; this structural motif is involved in IN multimerization; however, it makes the greatest contribution to the formation and stabilization of DNA complexes that are either specific to the sequence or nonspecific. A non-typical double signal of nuclear localization located in the catalytic ( ^186^ KRK ^188^ ) and C-terminal ( ^211^ KELQKQITK ^219^ ) IN domains is recognized by the participants of the importine/caryopherine cellular path. This interaction is enough to involve the PIC into the cell nuclear transport system [24].

**Table 1:** Results of *in vitro* and cell studies of diketo acid L-731.988 and naphthyridine derivatives L-870.810 and L-870.812 as HIV-1 integration inhibitors

Integrase inhibitor	IC_50_, in vitro(strand transfer), nM	CIC_95_ex vivo
L-731.988	8–15 [34]	CIC_50 _= 1 µM [34]
L-870.810	8–15 [35]	15 nM (10% FBS)100 nM (50% NHS) [36]
L-870.812	40 [36]	250 nM (50% NHS) [37]

The structure of full-scale HIV-1 IN remain unknown; only the structure of separate domains and double-domain IN fragments has been determined (see [20]). These data, along with the results obtained using site-directed mutagenesis and cross-linking, have been used to design computer models of IN [25–27]. Regardless of the fact that these models often contradict each other, most researchers share the opinion that the tetrameric form of IN is the one that functions in the cell. This viewpoint was confirmed by Hare *et al* . [28], who were the first to succeed in crystallizing and decoding the structure of retroviral IN in a complex with DNA. The IN of the human foamy virus belonging to the retroviruses from the *Spumaviridae* subgroup, which was used with this purpose in mind, is active in tetrameric form. A tetramer consists of asymmetrical dimers, each of those interacts with one terminus of viral DNA and performs its integration into cellular DNA [28]. The comparative study of the catalytic characteristics of the IN of the human foamy virus and HIV-1 revealed a considerable similarity in the functioning of these two enzymes [29, 30].

Both the cellular and viral proteins that are components of the preintegration complex can affect the catalytic activity of IN. HIV-1 IN needs neither viral nor cellular cofactors for the incorporation of both ends of the viral DNA into the super-spiralized cellular DNA [31]. However, it has been demonstrated that such proteins as the NC viral protein and cellular proteins HMG I(Y) and LEDGF/p75 can enhance the integration efficiency [21, 32]. It has been known that cellular protein LEDGF/p75 immediately interacts with IN and stimulates consistent integration and IN strand transfer [33]. It is assumed that LEDGF/p75 can play the role of the chaperone with respect to IN, stabilizing its multimeric organization, and enhancing the IN affinity towards DNA [21].

## Designing hiv-1 integration inhibitors suppressing the strand transfer reaction

**Fig. 3:**
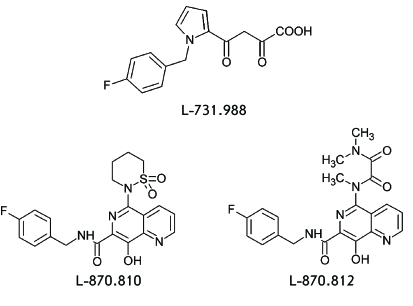
Structure of HIV-1 integration inhibitors: L-731,988, L-870,810, and L-870,812.

Since there has been no data available pertaining to the structure of HIV-1 IN, screening of libraries of chemical compounds of various classes has for a considerable period remained the primary method in the search for its inhibitors [4]. Testing of a library consisting of 250,000 compounds that ended by the year 2000 allowed specialists at Merck Pharmaceuticals (United States) to reveal a series of substances possessing the highest IN-inhibiting activity among them [34]. It appeared that all these substances are diketo compounds (DKC); notably, derivatives of 2,4-dioxobutanic acid. The inhibitors contained the so-called β-diketo acid motif, capable of coordinating cofactor metal ions in the IN active center [34]. These inhibitors manifested higher activity when inhibiting the strand transfer reaction than upon *in vitro* inhibition of the 3’ processing. The most active compound, L-731.988 ( *Fig. 3* ), was more active by a factor of 70 with respect to the strand transfer reaction in comparison with the 3’ processing reaction. Moreover, this inhibitor suppressed the development of HIV-1 in a cell culture ( *Table 1* ).

The selection of virus strains stable towards the action of DKC was performed, followed by the determination of the mutation site. Replacement of the М154 (M154I) residue located in the immediate proximity of the E152 residue (a component of the catalytic triad of the enzyme) was found to exist in the IN of a strain resistant to the action of L-731.988 [34].

The interaction between L-731.988 and IN has been subsequently studied [38]. It has been demonstrated that the inhibitor, at concentrations up to micromolar ones, does not interact with the isolated enzyme. A DNA substrate, U5 or U3-terminal fragments of the viral DNA are required for its binding with the enzyme. The dissociation constant ( *K*_d_ ) of the integrase–L-731.988 complex determined in the presence of 100 nM of U5 substrate was equal to 75 nM, which correlated with the IC _50 _ value in the strand transfer reaction. The affinity of L-731.988 upon interaction with IN bound to the processed viral DNA was higher by a factor of 100 in comparison with that upon the interaction with IN, in the absence of viral DNA ( *K*_d_ = 10–20 µM). The random sequence DNA did not stimulate interaction between the inhibitor and the enzyme. Furthermore, an increase in the concentration of the DNA substrate, its excess amount being capable of acting as a DNA target, resulted in a decrease in inhibitor–IN binding. An assumption was made, based on these data, [38] that the L-731.988 inhibitor suppresses the strand transfer reaction by competing with a DNA target for its binding site; the conformation of the active enzyme–substrate complex is required for the interaction between the inhibitor and IN.

A number of other substances have a similar nature of inhibition of the IN activity; their common feature being the presence of two oxygen atoms capable of coordinating cofactor metal ions in the IN active center [39–42].

The search for DKS analogues demonstrating anti-integrase activity resulted in the design of\ naphthyridine derivatives by Merck Pharmaceuticals. The activity and selectivity of these compounds with respect to the strand transfer reaction was similar to that of DKC [43]. Among the inhibitors of this structural class, two inhibitors, L-870.810 and L-870.812, have been subjected to the most thorough study ( *Fig. 2, Table 1* ).

A naphthyridine derivative L-870.810 displaced diketo acid L-731.988 from the IN/DNA-substrate complex, which enabled one to assume the possibility of binding of these compounds to IN at a single or overlapping site [36]. The accumulation of circular viral DNA in the treated L-870.810 cells and a decrease in the amount of integrated viral DNA served as evidence of the action of this inhibitor on the integration process. The selection of virus strains that could be resistant towards the action of L-870.810 resulted in the following substitutions in the primary structure of IN: F121Y/T125K, V72I/F121Y/T125K, and V72I/F121Y/T125K/V151I [36]. The viruses carrying the corresponding mutations were less sensitive to the action of L-870.810 by a factor of 4–100 in comparison with wild-type HIV-1 [36]. Mutations in the IN gene causing viral resistance to diketo acids and naphthyridines are closely located, but not identical. All these facts attest to the possibility of the existence of both a common binding centre and a common mechanism of action in inhibitors belonging to both classes.

Inhibitor L-870.810 successfully passed the first phase of clinical trials; however, at the second phase it proved toxic to kidneys and the liver. For this reason, the trials were ended.

## RALTEGRAVIR (MK-0518) – the first inhibitor of hiv-1 integration allowed for use

**Designing raltegravir**

**Fig. 4:**
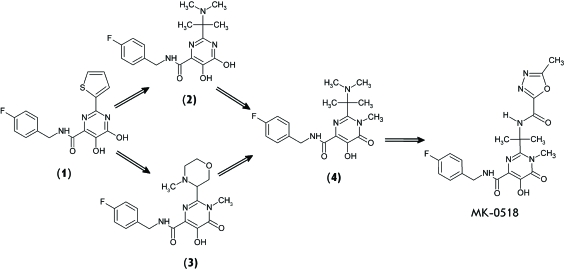
Optimization of dihydroxypyrimidine (1, 2) and *N* -methyl pyrimidinone carboxamides (3, 4) as HIV-1 integrase strand transfer inhibitors, which led to the design of raltegravir (inhibitor MK-0518).

The relative success achieved from the use of naphthyridine derivatives as IN inhibitors led to the design of inhibitors based on dihydroxypyrimidine (compound ( **1** ), *Fig. 4* ) [45]. This compound specifically suppresses the strand transfer carried out by recombinant IN ( *Table 2* ); however, even micromolar concentrations of this compound are inactive in the culture of infected cells. Nevertheless, due to its pharmacokinetic indices determined in rats (good bioavailability (F = 39%) and the low clearance of blood plasma ( *Cl*_p _ = 11 mg/min/kg)), this compound was selected for further structural and functional studies [45], enabling the design of this compound **(2)** *(Fig. 4) * [46]. The compound successfully inhibits the strand transfer reaction ( *Table 2* ) and suppresses the cytopic effect of HIV-1 in infected cells [46].

 Parallel studies resulted in the design of a novel class of IN inhibitors, N-alkyl pyrimidinone derivatives, which inhibited the nanomolar concentrations of IN in *in vitro* experiments [36]. The results of the study of the inhibiting effect of compound **(3)** *(Fig. 4), * belonging to this class, are listed in *Table 2* [47]. Moreover, compounds ( **2** ) and ( **3** ) were characterized by a strong pharmacokinetic profile and strong bioavailability in preclinical trials performed on rats, dogs, and rhesus macaques [46, 47].

An attempt was then made to combine the optimal properties of inhibitors from each of the two series in one molecule [48]. This approach resulted in the design of another N-methyl pyrimidinone derivative (compound ( **4** ), *Fig. 4* ). However, compound ( **4** ) turned out to be less active ( *Table 2* ).

The structural and functional studies of N-methyl pyrimidinone derivatives as IN inhibitors were continued [48]. Compound MK-0518 ( *Table 2* ), which was given the name ‘Raltegravir’ ( *Fig. 4* ), appeared to be the most active. It turned out that raltegravir has a high selectivity with respect to IN and has almost no inhibiting effect (IC _50_ > 50 µM) on such Mg ^2+^ -depending enzymes as HIV-1 reverse transcriptase, HIV-1 RNAse H, hepatitis C virus RNA polymerase, and human polymerases α, β, and γ [48]. No effect of raltegravir (at concentrations of up to 10 µM) on another 150 different enzymes, receptors, and channels has been revealed. In particular, raltegravir has no effect on various cytochrome P450 isoforms (IC _50_ > 50 µM) and does not bind to the hERG ion channel [48].

**Pharmacokinetics and interaction with HAART components**

**Table 2:** Results of *in vitro* and cell studies of derivatives of dihydroxypyrimidine and N-methylpyrimidinone and raltegravir (MK-0518) as HIV-1 integration inhibitors

Compound	IC_50_,in vitro(strand transfer), nM	CIC_95_ex vivo	Reference
(1)	10	inactive	[45]
(2)	50	60 nM (10% FBS)78 nM (50% NHS)	[46]
(3)	60	60 nM (10% FBS)100 nM (50% NHS)	[47]
(4)	250	1 µM (10% FBS)> 1 µM (50% NHS)	[48]
MK-0518	15	19 nM (10% FBS)31 nM (50% NHS)	[48]

The pharmacokinetic profile of raltegravir has been studied on rats, dogs, and rhesus macaques [48]. The rats were given raltegravir in three different forms (OH form, as well as Na ^+^ and К ^+^ salts) at a dose of 3 mg/kg. The best results were achieved when using raltegravir salts. The amount of the compound that entered the blood upon one-time administration (AUC) was the highest for Na ^+ ^ salt; the maximum peak of compound concentration in blood ( *С*_max_ ) was attained in K ^+ ^ salt. In this case, values AUC and *С*_max _ were also better in the salt form of the preparation ( *Table 3* ). The dogs were given raltegravir in the form of the OH form or K ^+ ^ salt at a dose of 10 mg/kg. In this case, AUC and *С*_max_ values were also better for the salt form of the drug ( *Table 3* ). The crystalline OH form at a dose of 10 mg/kg was also administered to rhesus macaques, but the AUC and *С*_max _ indices were quite low ( *Table 3* ). The bioavailability of the drug administered orally ( *F* ) also appeared to be better for the K ^+^ salt in comparison with that for the OH form ( *Table 3* ). Hence, the pharmacokinetic profile of raltegravir in the form of Na ^+^ and К ^+ ^ salts was found to be preferable to that of the OH form; salt forms being characterized by improved solubility [48].

Blood plasma clearance was appreciably low in dogs and was characterized by medium values in rats and rhesus macaques ( *Table 3* ). Furthermore, raltegravir binding to the blood plasma proteins (PPB) of rats, dogs, and rhesus macaques has been the subject of a study ( *Table 3* ) [48], as well as its metabolism in the liver microsomes of these animals and humans. It appeared that the drug is metabolized by glucuronosyltransferase [48]. The same conclusion was made after studying the metabolism in hepatocytes [48] and was borne out by the results of an independent study [49]. ^1^ Н- and  ^13^ С-NMR spectroscopy was used to completely characterize the formation of a conjugate between glucuronide and a hydroxylic group at position 5 of the pyrimidinone ring of raltegravir [48].

**Table 3:** Pharmacokinetic parameters of raltegravir determined for rats/dogs/rhesus macaques [48]

Form	AUC, µM·h	C_max_, µM	T_1/2_, h	*F*, %	Cl_p_, ml/min/kg	PPB, %
OH	1.0/21/1.8	1.2/8/0.3	ND*/ND/7	37/45/8	39/6/18	74/71/85
Na^+^	1.4/ND/ND	1.0/ND/ND	ND/ND/ND	ND/ND/ND	ND	ND
K^+^	1.3/45/ND	1.6/24/ND	73/13/ND	45/69/ND	ND	ND

* ND – no data available.

The comparison of *in vivo * and * in vitro * data allowed one to suppose [48] that the pharmacokinetic profile of raltegravir for humans will be similar to the drug profile for a dog.

To achieve a therapeutic effect, it is necessary that the drug concentration 12 h after the administration (С _12_ ) remain above CIC _95_ = 31 nM. With account for the data on raltegravir binding with blood plasma proteins, metabolic stability, half-excretion period, and clearance, peroral administration of raltegravir К ^+^ salt at a dose of at least 100 mg twice a day was proposed during the clinical trials.

The pharmacokinetic profile of raltegravir was determined both in healthy volunteers and HIV-infected patients. Two randomized placebo-controlled trials were carried out with healthy volunteers: 32 volunteers were given a single dose of raltegravir (10–1600 mg); and raltegravir was administered to 40 volunteers every 12 h for a duration of 10 days (100–800 mg) [50]. It was ascertained that raltegravir is characterized by a good assimilability; its content in blood plasma attaining the maximum level ( *С*_max_ ) as early as after 1 h. The half-excretion period of the drug ( *Т*_1/2_ ) was equal to 7–12 h. The drug concentration in the blood becomes constant as early as 2 days after its administration; after the administration is stopped, only weak accumulation in the organism is observed [50]. Moreover, it should be noted that no considerable differences were revealed to administration of the drug in the male or female volunteers.

The pharmacokinetics of raltegravir was also studied among HIV-infected antiretroviral treatment-naïve patients [51]. It was ascertained that the AUC and  *С*_max _ values increase in geometrical progression up to the administration of raltegravir, at a dose of 400 mg, twice a day. At a dose of 600 mg, these parameters do not increase [51]. Moreover, an approximately threefold decrease in the viral load in HIV-infected patients was observed, regardless of the dose of the administered drug [51]. Nevertheless, the latter results should be taken with great care due to the relatively small size of the sampling and short duration of the study.

The interaction of raltegravir with various components of HAART antiviral therapy in healthy volunteers has also been studied (see [52]). It appeared that the simultaneous administration of raltegravir and protease inhibitors – atazanavir and atazanavir/ritonavir mixture – increases raltegravir concentration in the blood to a certain extent, whereas ritonavir alone has almost no effect on the raltegravir concentration. The nucleotide inhibitor of reverse transcriptase, tenofir, also had a negligible effect on the raltegravir concentration. The administration of raltegravir, together with tipnavir (protease inhibitor), maraviroc (penetration inhibitor), efavirenz, and etravirine (both drugs are non-nucleoside inhibitors of reverse transcriptase), resulted in a decrease in the raltegravir concentration in healthy volunteers.

Only one study of raltegravir interaction with HAART components has been carried out on HIV-infected patients [53]. Four individuals were given raltegravir (400 mg) twice a day, together with etravirine (non-nucleoside inhibitor of reverse transcriptase); the raltegravir concentration in the blood decreased by a factor of 4. Regardless of these results, the authors provide no recommendations on changing the dose of raltegravir when administering it together with etravirine [53]. Thus, the necessity for further study of the interaction between raltegravir with HAART components in HIV-infected individuals becomes evident. 

**Clinical trials**

*Treatment upon resistance to HAART. *Firstly, the study of raltegravir in HIV-infected individuals who had received HAART and acquired resistance to its components was initiated. The randomized double-blind, placebo-controlled trial (P005) was carried out during 24 weeks in research centres within the United States, Europe, Latin America, and Asia [54]. The participants included adult patients (18 years and older) with a viral load of at least 5,000 HIV RNA copies/ml and with a level of CD ^+ ^ lymphocytes of at least 50 cells/µl. The patients had also been receiving HAART on a regular basis for at least 3 months, and had laboratory-confirmed genotypic or phenotypic resistance to at least one of the non-nucleoside inhibitors of reverse transcriptase, one nucleoside inhibitor of reverse transcriptase, and one protease inhibitor. Prior to the random grouping, the basic regimen of HAART was optimized for each patient; the amount of drugs administered varying from two to seven. It should be noted that the selection of antiretroviral agents for these patients was very limited due to the intolerance or HIV-1 resistance to them. Since it had been demonstrated earlier that the simultaneous administration of atazanavir (protease inhibitor) and raltegravir may result in an increase in raltegravir concentration in the blood [50], the patients were divided into two subgroups, those with atazanavir included within their basic regimen, and those without it [54].

**Table 4:** Results of clinical trials of raltegravir

Name and duration of the trial	Therapeutic strategy	Fraction of patients with an undeterminable viral load (< 50 copies/ml)	CD4 cell count, cells/µl	Reference
Therapy upon HAART-resistance				
P005, 24 weeks	200 mg ral.*, 41 h	65	63	[54]
	400 mg ral., 44 h	56	113	
	600 mg ral., 45 h	67	94	
Placebo, 45 hhh	13	5		
BENCHMRK-1 and -2, 96 weeks	400 mg ral., 462 h	58	123	[55]
Placebo, 237 hhh	26	49		
Primary therapy				
P004, 4 weeks	100 mg ral., 41 h	85	221	[56]
	200 mg ral., 40 h	83	146	
	400 mg ral., 41 h	88	144	
	600 mg ral., 40 h	88	187	
600 mg efv.*, 39 h	87	170		
STARTMRK-1 and -2, 48 weeks	400 mg ral., 281 h	86	189	[57]
600 mg efv., 282 h	82	163		
Supporting therapy				
CHEER, 24 weeks	400 mg ral., 52 h	94	32	[58]

* ral. – raltegravir.

** efv. – efavirenz.

The trial comprised 175 patients. All patients were divided into groups, which were either given varying doses of raltegravir or a placebo ( *Table 4* ). The clinical and demographic characteristics were similar for the groups. All patients underwent a 24-week therapy course. Raltegravir as a supplement to the optimized HAART regimen showed better efficacy in comparison with the placebo, at any given dose. A mean decrease in the viral load by 100 copies/ml was observed in all groups who were given raltegravir; it began as early as week 2 and was consistent up to week 24 ( *Table 4* ). The decrease in the viral load was accompanied by an increase in the CD4 ^+^ cell count ( *Table 4* ). Joint use of raltegravir and enfuvirtid (entry inhibitor) or atazanavir within HAART particularly improved the virological and immune response. Forty-one patients left the trial because of inefficacy, 14 (11%) of the 144 who were given raltegravir and 27 (60%) of the 45 who received a placebo. It was demonstrated that raltegravir is a very safe drug: most of the side effects were of light or medium degree of severity. Only two patients left the trial as a result of the side effects of HAART (one in the raltegravir group, and one in the placebo group) [5 4].

The results of the P005 trial [54] in general are consistent with the results of the long-term trial BENCHMRK-1 and -2 [55] devoted to the study of the efficacy and safety of using raltegravir in patients who had earlier received HAART. After a 96-week study, it was found that the viral load had decreased and the CD4 cell count had increased quite considerably in patients who were given raltegravir than it had in those who received a placebo ( *Table 4* ). It should be mentioned that the optimum results were achieved when raltegravir was used in combination with darunavir (protease inhibitor) and enfuvirtid; the patients were naive to these drugs [55].

*Primary therapy*. Since raltegravir demonstrated positive results in patients who had earlier received HAART, it became attractive as a primary drug for HIV-infected patients naïve to HAART. The effect of raltegravir and efavirenz (non-nucleoside inhibitor of reverse transcriptase) were compared in two series of clinical trials (P004 [56] and STАRTMRK-1 and -2 [57]). As a supplement to these drugs, the participants were given tenofovir and lamivudin (both drugs are nucleoside inhibitors of reverse protease) (P004 [56]) or tenofovir only (STARTMRK-1 and -2 [57]). It turned out that raltegravir was no less capable of reducing the viral load to below 50 copies/ml in comparison with efavirenz; the CD4 cell count being comparable for both drugs ( *Table 4* ). In addition, the therapeutic effect of raltegravir was observed earlier than that of efavirenz [57]. Although the reasons for such an effect have as yet remained unclear, this property of raltegravir can be potentially used when there is a necessity for a rapid decrease in the viral load after the HIV-infection, or to reduce the risk of transplacental infection of the foetus from a HIV-infected mother. It should also be noted that in general the results demonstrated in trials P004 and STАRTMRK-1 and -2 were better than those in trials Р005 and BENCHMRK-1 and -2 ( *Table 4* ). This result posits the early commencement of raltegravir administration to HIV-infected patients. 

*Supporting therapy*. In addition to using raltegravir as a therapeutic agent in HAART-naive patients, as well as in patients who had developed resistance to HAART components, it was proposed to substitute antiretroviral drugs with raltegravir in patients with a viral load that cannot be determined, in order to reduce the side effects. Trial СHEER [58] included 52 patients with a viral load of less than 50 copies/ml, who were earlier given enfuvertid (penetration inhibitor). Twenty-four weeks after transferring to raltegravir, the undeterminable viral load remained in 49 patients ( *Table 4* ). Based on these results, a conclusion can be drawn that the substitution of enfuvertid by raltegravir seems to be appreciably safe.

**Safety**

How safe Raltegravir is was evaluated both in HIV-infected patients naïve to HAART and in patients who developed resistance to HAART components. All studies demonstrated a strong degree of drug tolerance [51, 56, 57]. Raltegravir turned out to be safer than efavirenz upon primary therapy of HIV-infected patients [57]. How safe raltegravir is was assessed on patients who had earlier received HAART. Recurrent or progressing cancer types were detected in approximately 3.5% of patients who were administered raltegravir, whereas this index was equal to 1.7% in individuals taking a placebo. Such types of cancers as Kaposi’s sarcoma, lymphoma, hepatic cancer, etc. were the most common [54]. The thorough analysis of all cases of cancer development in patients who were administered raltegravir revealed no correlation between drug administration and the emergence of malignant tumors [52].

**Usage guidelines**

Raltegravir successfully passed all phases of clinical trials in October 2007 and was approved by the FDA as a therapeutic agent for patients with resistance to HAART components [1]. In July 2009, the FDA also authorized the use of raltegravir for primary therapy of HIV-infected patients [59]. Raltegravir was registered under the trademark Isentress ^TM^ ; it is manufactured in the form of 400 mg tablets for twice-daily oral administration.

**Development of resistance to raltegravir**

Raltegravir has been used with appreciable success as one of the components of HAART; however, a number of patients developed resistance to this drug [60]. It was ascertained during the trial BENCHMRK-1 and -2 that 48 weeks after raltegravir administration commenced, resistance to this drug was developed in approximately 25% of patients. Virus isolates were obtained from 94 patients with resistance. No mutations in the IN genes were found in 30 isolates; whereas in the remaining 64, the development of resistance was accounted for by these mutations [60]. The resistance to strand transfer reaction is usually associated with the mutations in the IN active center [61]. It was in the active center that raltegravir-resistant virus isolates contained the primary mutations Y143R/C, Q148K/R/H, and N155H [60]. In most patients (48 out of 64), the virus had at least two mutations. Typically, it was a primary mutation and one or several secondary mutations. The primary mutation Y143R/C was associated with the secondary mutations L74A/I, E92Q, T97A, I203M and S230R; the mutation Q148K/R/H was associated with mutations G140S/A and E138K. Primary mutation N155H was associated with a number of secondary mutations: L74M, E92Q, T97A, V151I, and G163R [60]. In addition, it was ascertained that it is typical for mutations to accumulate with time. At the first instance, it refers to the Q148R substitution, which renders the virus almost unsusceptible to raltegravir. The fraction of carriers of the virus containing this mutation among raltegravir-resistant patients after undergoing therapy for 48 weeks was equal to 27%; after 96 weeks, the percentage increased to 53%. Meanwhile, the fraction of carriers of the virus with the N155H mutation shrank from 45 to 18% [60]. The probability of emergence of raltegravir-insusceptibility decreased in patients with reduced viral load (< 100,000 copies/ml) and in patients who were administered other active antiretroviral drugs.

A similar pattern was revealed in trial P005 [54]. In 20 and 14 patients out of 35 who appeared to be unsusceptible to raltegravir, the virus contained mainly the Q148K/R/H or N144H mutation, respectively. Mutations N155H and Q148K/R/H reduced the sensitivity of HIV patients to the action of raltegravir by factors of 10 and 25, respectively. Similar to that in trial BENCHMRK-1 and -2, mutation Q148K/R/H turned out to be associated with the secondary substitutions E138K and G140S/A. The secondary mutations L74M, E92Q, and G163R were revealed, and no substitutions T97A and V151I were found in the case of N155Н. The probability of resistance development decreased upon reduced viral load and when using additional HAART active components [54].

**Fig. 5:**
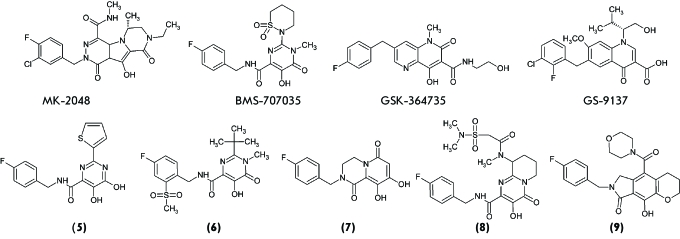
Structures of HIV-1 integrase strand transfer inhibitors: MK-2048, BMS-707035, GSK-364735, GS-9137, and some new inhibitor classes: dihydroxypyrimidines (5), N-methyl pyrimidinone carboxamides (6), dihydroxypyrido-pyrazine-1,6-diones (7), bicyclic pyrimidones (8), pyrrolloquinolones (9).

The recently obtained data in study [62] in which the effect of raltegravir on the strand carrier reaction performed by wild-type IN or IN containing a mutation G140S, Q148H, or a double mutation G140S/Q148H are also of interest. It was evident that while the G140S mutation results in the emergence of small resistance (IC _50_ (WT) = 10 nM, IC _50_ (G140S) = 30 nM), IN with the Q148H or G140S/Q148H mutations has a very high raltegravir resistance (IC _50_ (Q148H) > 70 nM, IC _50_ (G140S/Q148H) > 1000 nM). In addition, it was ascertained that the G140S mutation in the IN gene recovers the weak infectivity of the virus with the Q148H mutation to the level of the wild-type virus [62].

## RALTEGRAVIR ANALOGUES – inhibitors of HIV-1 Integration

The emergence of a new drug, as in the case of any other kind of innovation, results in the rapid appearance of a number of its analogues. Taking into account the approximate cost of the development, trials, and implementation of a single drug, which is equal to $ 2 million, together with the fact that only one out of three novel therapeutic agents manage to recoup this expenditure [18], it is clear that the temptation exists for pharmaceutical companies to avoid the difficulties associated with the development of a completely new drug and confine themselves to modifying the drug that is the best for the moment. Therefore, raltegravir, being the only licensed inhibitor of HIV integrase so far, is of immense interest as a starting point in the development of integration inhibitors. Raltegravir analogues are usually compounds based on diketo acids, which specifically suppress the IN strand transfer reaction due to chelating of Mg ^2+^ ions in the enzyme active center [18]. In this section, we shall turn our attention to the inhibitors of strand transfer eligible for the phase of clinical trials.

**MK-2048**

Soon after the permission for the use of raltegravir as a therapeutic agent was obtained, Merck Pharmaceuticals attempted to design pharmacophore, typical of diketo acids and capable of interacting with the metal ion on the basis of tricyclic dihydroxypyrrole derivatives [63]. As a result, the MK-2048 inhibitor was designed ( *Fig. 5* ), which exhibited a high inhibition activity in all experiments ( *Table 5* ). The inhibitor has a good pharmacokinetic profile and, more importantly, possesses potential activity with respect to four mutant IN forms that are resistant to raltegravir. МК-2048 is currently undergoing clinical trials [18].

**BMS-707035**

**Table 5:** Results of *in vitro* and cell studies of raltegravir analogues as HIV-1 integration inhibitors

Compound	IC_50_, in vitro(strand transfer), nM	CIC_95_ex vivo	Reference
MK-2048	10	35 nM (50% NHS)	[63]
BMS-707035	20	-	[16]
GSK-364735	8	EC_50_= 1.2 nMEC_90_= 42 nM (20% NHS)	[68]
S/GSK 1349572	2.7	EC_50_= 0.5 nMEC_90_= 2 nM	[13]
GS-9137 (elvitegravir)	7	EC_50_= 0.7 nMEC_90_= 1.7 nM (20% NHS)	[72]
(5) (dihydroxypyrimidine)	10	> 10 µM (10% FBS)	[48]
(6) (N-methylpyrimidinone)	20	10 nM (10% FBS)20 nM (50% NHS)	[64]
(7) (dihydroxypyrido-pyrasine-1,6-dion)	100	310 nm (10% FBS)310 nM (50% NHS)	[65]
(8) bicyclic pyrimidinone	7	16 nM (10% FBS)31 nM (50% NHS)	[66]
(9) pyrrolquinolone	13	7 nM (10% FBS)16 nM (50% NHS)	[67]

The structural and functional motifs of inhibitors L-780.810 and raltegravir are combined in the structure of this inhibitor [16]. BMS-707035 differs from raltegravir only by the substitution of the oxadiazole group for the cyclic sulfonamide group ( *Fig. 5* ) and possesses *in vitro* inhibiting activity that is similar to that of raltegravir ( *Table 5* ). BMS-707035 has reached the second phase of clinical trials; however, multiple mutations emerged in the IN gene responding to the therapy, which has led to the emergence of resistant HIV strains [16]. In the beginning of 2008, the clinical trials of BMS-707035 were discontinued.

**GSK-364735**

The GSK-364735 inhibitor ( *Fig. 5* ), a naphthyridinon derivative [68], was developed by the merged Shionogi-GlaxoSmithKline Pharmaceuticals company on the basis of one of the first inhibitors of strand transfer S-1360 [42]. It was efficient in the suppression of HIV replication in MT-4 cells ( *Table 5* ) and possessed a low cytotoxicity (СС _50_ > 10 µM). The investigation of the action of GSK-364735 on HIV strains containing mutations in the IN gene has demonstrated that the inhibitor is more active to a certain extent with respect to the viruses with mutations T66I (by a factor of 1.2), E92Q (by a factor of 3.7), P145S (by a factor of 1.4), Q146R (by a factor of 1.7), and Q153Y (by a factor of 1.4) as compared with its activity towards the wild-type virus. However, a considerable reduction in the activity of the GSK-364735 inhibitor was observed in the case of four main mutations in the gene of HIV-1 integrase, which result in resistance development: by factors of 17 (T66K mutation), 210 (Q148K mutation), 73 (Q148R mutation), and 23 (N155H mutation) [68]. It was ascertained at the preclinical research phase that GSK-364735 has an acceptable pharmacokinetic profile; the bioavailability indices *F* (42, 12, and 32%), the half-excretion period from blood plasma *T*_1/2_ (1.5, 1.6, and 3.9 h), and plasma clearance *Cl*_p_ (3.2, 8.6, and 2 ml/min/kg) were obtained on rats, dogs, and rhesus macaques, respectively [68]. The drug had good indices at the first phase of clinical trials: it was ascertained that GSK-364735 is capable of reducing the viral load by a factor more than 100. However, the clinical trials ceased at the second phase due to hepatotoxicity being revealed [68, 69].

**S/GSK1349572**

Shionogi-GlaxoSmithKline Pharmaceuticals has reported that they have designed a highly efficient inhibitor S/GSK1349572 ( *Table 5* ) [70]. The authors have not disclosed the structure of this compound, but they claim that the agent is capable of specific inhibition of the strand transfer reaction; the mechanism of its action being based on chelating of Mg ^2+^ ions in the active centre of the IN [70]. These facts allow us to tentatively attribute S/GSK1349572 to raltegravir analogues, if not in terms of structural characteristics, then at least on the basis of its effect on IN. The use of this preparation results in the development of mutations in the IN gene; however, they are incapable of providing a high degree of virus resistance to S/GSK1349572. Interestingly, the inhibitor turned out to be active with respect to the HIV strains that were resistant to raltegravir [70] and elvitegravir (see below). This drug is likely to have a different resistance profile [14]. Nevertheless, certain secondary mutations that are additional to G140S/Q148H, such as T97A, M154I or V201, induce resistance both to S/GSK1349572 and raltegravir [71]. These data point to the necessity of subjecting the emergence of HIV-1 resistance to this inhibitor to further study.

It was established by studying healthy volunteers that S/GSK1349572 has a rather positive pharmacokinetic profile; in particular, its bioavailability upon peroral administration was approximately 70%, its period of half-excretion from blood plasma *Т*_1/2 _ being higher than 15 h [70]. At the time of writing, the second phase of clinical trials of S/GSK1349572 is in progress [70].

**GS-9137 (elvitegravir)**

The attempts to modify DKS pharmacophore made byJapan Tobacco (Japan) resulted in the design of a group of IN inhibitors based on 4-oxoquinoline, which retained the arrangement of the major functional groups that are required for the interaction with metal ions [72]. The cooperation agreement between Japan Tobacco and Gilead Sciences (United States) signed in 2005, laid the foundation for the clinical trials of the GS-9137 inhibitor ( *Fig. 5* ) [16] named elvitegravir, as well as the most active representative of IN inhibitors belonging to this structural class ( *Table 5* ). The pharmacokinetic profile of elvitegravir was studied on rats and dogs [73]. The drug had good indices of bioavailability *F* (34 and 30%), period of half-excretion from blood plasma *Т*_1/2_ (2.3 and 5.2 h) and plasma clearance *Cl*_p _ (8.3 and 17 ml/min/kg) in rats and dogs, respectively [73].

The pharmacokinetic profile of elvitegravir was studied in both healthy [74] and HIV-infected [75] volunteers. It was established that elvitegravir rapidly assimilates (3.5–4 h); an increase in *С*_max_ and AUC parameters was observed with an increasing elvitegravir dose. The best results were obtained when administering elvitegravir together with ritonavir (protease inhibitor) [75]. Unlike raltegravir, elvitegravir is metabolized by cytochrome P450 (CYP3A4) [74]. The stimulating action of ritonavir is probably the result of its ability to inhibit Р450 cytochrome and thus maintain a higher concentration of elvitegravir. The interaction between elvitegravir and nucleoside and non-nucleoside inhibitors of reverse transcriptase and penetration inhibitor maraviroc was also studied. It turned out that these inhibitors have no considerable effect on the efficacy of elvitegravir [74].

A randomized study of the therapeutic activity of elvitegravir was performed on 278 HIV-infected patients who had earlier received HAART and developed resistance to its components [76]. The patients with a viral load of approximately 30,000 copies/ml and CD4 lymphocyte count of approximately 200 cells/µl were given the combination of elvitegravir and ritonavir once per day. After week 24 of trial, the viral load decreased by a factor of at least 10 in 90% of the patients who had been administered elvitegravir. The viral load decreased by a factor of 100 in 76% of the individuals who were given 125 mg of elvitegravir and 69% of those who were administered 50 mg of elvitegravir [76]. The study was also performed on 40 HIV-infected volunteers with a viral load of 10,000–300,000 copies/ml and an average CD4 cell count of approximately 200 cells/µl, who received elvitegravir at different doses once or twice a day or once a day in combination with ritonavir. The viral load decreased on average by a factor of 80 in the groups administered elvitegravir twice a day and those administered the combination of elvitegravir and ritonavir, after 10 days. However, no statistically significant change in the CD4 cell count has been observed [75].

A randomized double-blind study of the effect of elvitegravir on HIV-infected volunteers who were naïve to HAART was carried out for 48 weeks [77]. The patients tested were divided into two groups: one of those received a mixture of elvitegravir with cobicistat, the inhibitor of cytochrome Р450; the second group was given the non-nucleoside inhibitor of reverse transcriptase efavirenz. In addition, both groups were administered two nucleoside inhibitors of reverse transcriptase, emtricitabine /tenofovir. It appeared that the fraction of patients with an undeterminable viral load in both groups reached 83% and 90% after weeks 24 and 48, respectively [77]. Thus, elvitegravir manifested a high efficacy, which was comparable with the efficacy of the commonly used antiviral agent efavirenz.

The data on the safety of elvitegravir is limited by the results of the second phase of trials, in which HIV-infected patients were given an elvitegravir/ritonavir mixture or a competitive protease inhibitor [76]. No noticeable differences between the two groups in terms of either the frequency of side-effects, or their severity, was observed. Additional studies are required to confirm how safe elvitegravir is in the treatment of HIV-infected patients. At the time of writing, elvitegravir is undergoing the third phase of clinical trials.

Analysis of resistance development in the individuals administered elvitegravir is confined to the data obtained using HIV isolates collected from the patients participating in the second phase of clinical trials [52, 78]. Primary mutations E92Q, T66I/A/K, E138K, S147G, Q148R/H/K, and N155H in HIV-1 integrase were the most frequent; they are also associated with resistance to other DKC-based inhibitors, primarily to raltegravir [79]. At least one of the primary mutations was detected in 39% of elvitegravir-resistant individuals. In addition, cross-resistance to raltegravir was detected: on average, a decrease in the susceptibility to elvitegravir by a factor of 150 also resulted in a 30-fold reduction of susceptibility to raltegravir. The cross-resistance of HIV to the action of raltegravir and elvitegravir was verified by the results of cell studies [80]. Elvitegravir-resistant HIV strains were isolated, and increased resistance to raltegravir and the derivatives of diketo acid L-731.988 and naphthyridine L-870.810 was detected [80].

**New raltegravir-based inhibitors of HIV-1 integration**

**Fig. 6:**
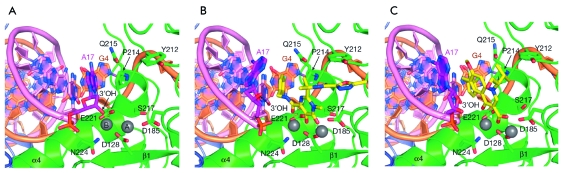
Structure of the active site of human foamy virus integrase in the absence of inhibitor (A), in the presence of MK-0518 (B), and GS-9137 (C) [28].

The conduct of a large number of studies aimed at searching for new raltegravir-based IN inhibitors resulted in the development of IN inhibitors belonging to several novel structural classes. They all specifically inhibit the strand transfer reaction and contain functional groups that are capable of chelating metal ions in the IN active centre [18]. The names of the new structural classes of IN inhibitors, the structure of individual representatives ( *Fig. 5* ) with the indication of their activity *in vitro* and in cell studies, and the data on studying pharmacokinetics in rats are presented in *Table 5.*

**The problems of usage of raltegravir analogues as integration inhibitors**

All the recorded data point to the fact that the known raltegravir analogues act upon HIV-1 integrase via the same mechanism, contain a similar structural motif, and manifest comparable activity * in vitro * and in cell studies. This type of identity casts some suspicion on the successful future application of these inhibitors as therapeutic agents. These suspicions are caused by cross-resistance of the virus to these inhibitors.

First of all, the emergence of cross-resistance can be accounted for by the similar mechanism of binding of strand transfer inhibitors to the IN complex and viral DNA [14]. As a result of the manner of binding of these compounds, they “push” the 3’-terminal hydroxyl of the processed DNA strand out of the enzyme active centre, thus blocking the integration. This binding mechanism was proposed for HIV-1 IN [81] and demonstrated for the enzyme of the human foamy virus ( *Fig. 6* ) [28]. Study [28] was first to show that the position of 3’-hydroxyl is occupied by the fluorobenzyl residue of the inhibitor, the fundamentally necessary structural element of all strand transfer inhibitors ( *Figs. 4 and 5* ).

Using data on X-ray diffraction of the catalytic domain of IN in its complex with Mg ^2+ ^ (1BL3) [18], molecular docking of certain IN inhibitors based on DKC was performed. According to the model proposed, raltegravir interacts with the T66, E92, Y143, Q148, and N155 residues, their substitution resulting in a decrease in the susceptibility to raltegravir by a factor varying from 5 to 35 [48, 82]. Molecular docking also confirmed the fact that amino acid residues interacting with GSK-364735 and GS-9137 are identical to those interacting with raltegravir [83, 84], with an exception for G140. For the latter, a similarity is observed only in the case of elvitegravir [18]. This result correlates with the data demonstrating that the G140S mutation reduces HIV-1 susceptibility to elvitegravir by a factor of 4; and that to raltegravir, only by a factor of 1.6.

Molecular docking of certain new IN inhibitors ( *Fig. 5)* demonstrated that these compounds bind to IN in a manner similar to that of raltegravir. Hence, if the results of the study [18] are valid, it is rather unlikely that new inhibitors based on raltegravir will turn out to be active with respect to HIV strains that are raltegravir-resistant and, therefore, become an adequate substitution for it.

Another problem that could complicate the successful use of integration inhibitors is the scarcity of knowledge on IN polymorphism in various subtypes of HIV-1. Until recently, only one study had been performed in which the susceptibility of 137 clinical isolates to raltegravir had been tested. Sixty of those isolates did not belong to the B-subtype [85]. No differences were revealed. However, it was demonstrated in *in vitro* experiments that the IN of the C-subtype virus containing the E92Q/N155H mutation is more susceptible to raltegravir and elvitegravir by a factor of 10 as compared with the enzyme of B-subtype HIV-1 [86]. It has also been demonstrated that the mutations of the G140 residue occur less frequently in the CRF02 AG-subtype virus as compared with those in the B-subtype virus [87]. Data are available that raltegravir appears to be inefficient more frequently on individuals infected with a non-B-subtype virus [88].

## Alternative paths of inhibition of hiv-1 integration

**Fig. 7:**
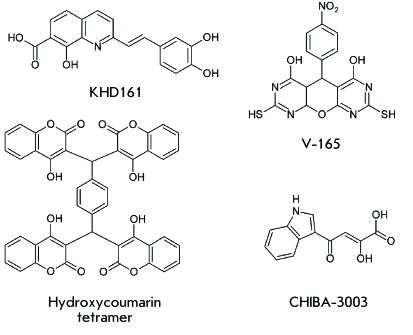
Structures of KHD161, V-165, hydroxycoumarin tetramer, and CHIBA-3003.

We believe that the optimal alternative way of searching for inhibitors of HIV-1 integration consists in designing inhibitors with a mechanism of action that differs from that of raltegravir and its analogues, which specifically inhibit the strand transfer. We shall provide only a brief characterization of several classes of IN inhibitors that differ from the inhibitors of strand transfer in terms of their mechanism of action, and specify certain representatives of these inhibitors.

**Inhibitors of 3’ processing**

The inhibitors belonging to this class are likely to suppress both integration stages: both 3’ processing and the strand transfer. It occurs due to the fact that they interact with the active centre of the enzyme, rather than with the enzyme–DNA substrate complex. It is an appreciably numerous class of inhibitors; styryl quinoline compounds being the best-studied inhibitors of 3’ processing [89]. Styryl quinoline KHD161 ( *Fig. 7* ) has nearly the same effect both on 3’ processing (IC _50_ = 2.4 µM) and the strand transfer (IC _50_ = 1 µM). It is capable of suppressing the cytopathic effect of HIV-1 in cells with CIC _50_ = 1.3 µM [90, 91]. IN inhibitors based on styryl quinoline are known to be incapable of destroying the pre-formed IN–DNA substrate complex and inhibiting the reaction of 3’ processing with its participation. Moreover, the ability of styryl quinolines to bind onto IN depends on Mg ^2+ ^ ions [92]. Thus, a competitive mechanism of IN inhibition with styryl quinolines, due to the interaction with a metal ion in its active centre, can be proposed.

**Allosteric inhibitors**

Inhibitor V-165 ( *Fig. 7* ), belonging to the class of 5 *H* -pyrano[2,3-d:-6,5-d’]dipyrimidines, prevents IN binding with the DNA substrate. It is more efficient in inhibiting the reaction of 3’ processing (IC _50_ = 0.9 µM), in comparison with that of the strand transfer (IC _50_ = 16 µM) [93]. Moreover, V-165 suppresses HIV infection in a cell culture [93]. A double mutation T206S/S230N in the IN gene was successfully identified by the selection of virus strains resistant to the inhibitor V-165, since the mutation is located in the C-terminal domain of IN, its main function consisting in DNA binding [94].

**Inhibitors of integrase multimerization**

The search for compounds with an effect on the interaction between the IN and components of the HIV-1 preintegration complex or on their own ability to form an active multimer is currently actively under way [95]. Hydroxycoumarin derivatives refer to the compounds that suppress IN multimerization. It has been demonstrated that hydroxycoumarin tetramer ( *Fig. 7* ) suppresses HIV-1 replication in a cell culture with the value * СIC*_50_ = 11.5 µM) [96]. It can inhibit the activity of HIV-1 integrase * in vitro* . Hydroxycoumarin tetramer inhibits 3’ processing and strand transfer (IC _50_ = 1.5–2.0 µM) [96]. The benzophenone derivative of hydroxycoumarin was crosslinked to integrase in order to identify the site of inhibitor–enzyme binding [97]. Peptide ^128^ AACWWAGIK ^136^ , to which the inhibitor binds, has been determined [97]. This peptide participates in the dimeric complex formation of the catalytic domain [39]. Thus, the hydroxycoumarin-based inhibitor is bound to the enzyme near the surface of contact between two monomers. The binding of hydroxycoumarin derivatives with IN near the ^128^ AACWWAGIK ^136^ peptide can disturb interaction of this kind and can have an effect on the formation and stability of the catalytically active integrase multimer. 

**Inhibitors of the interaction between integrase and LEDGF/p75**

This is the least explored direction in the search for integration inhibitors. Regardless of the fact that PIC contains a large number of viral and cellular proteins in addition to IN, it is its interaction between IN and its cellular partner LEDGF/p75 that determines HIV-1 integration [95]. Data on the inhibitors capable of destroying the IN/LEDGF complex is rather scarce; what we know so far is that their activity is likely to be low. The CHIBA-3003 compound ( *Fig. 7* ) was designed using a computer simulation. It is capable of destroying the IN/LEDGF complex with IC _50_ = 35 µM [98]. The effect of the LEDGF ^355^ IHAEIKNSLKIDNLDVRNCIEAL ^377 ^ peptide on the stability of the IN/LEDGF complex and catalytic activity of IN has been studied [99]. It appears that this peptide impedes the formation of the IN/LEDGF complex with IC _50_ = 25 µM and inhibits 3’ processing and strand transfer with IC _50_ = 160 µM [99].

## CONCLUSIONS

In 1996, the resources allocated to research, treatment, and prevention of the spread of HIV/AIDS amounted to 300 million USD. Since then, they have been steadily increasing, to approximately 10 million USD a year at the time of writing [18]. A considerable portion of these funds is spent on the development of new inhibitors aimed at the suppression of viral enzymes, including IN. During the 2.5 years that have elapsed since raltegravir was certified for use as a therapeutic agent against HIV-1, the major efforts of pharmaceutical companies such as Merck Pharmaceuticals, Japan Tobacco, Gilead Sciences, and Shionogi-GlaxoSmithKline Pharmaceuticals have focused on developing analogues of this drug. Yet, many raltegravir analogues are incapable of suppressing the replication of HIV-1 strains that are raltegravir-resistant [83, 84]. We consider that there is a need for a more active search for inhibitors with a different mechanism of action, which can be active with respect to the raltegravir-resistant viral strains. However, it should be mentioned that none of the inhibitors of HIV-1 integration that do not belong to the class of specific inhibitors of strand transfer has so far managed to successfully pass even the first phase of clinical trials. 

## Abbreviations

HAART: highly active antiretroviral therapy
HIV-1: human immunodeficiency virus type 1
IN: integrase
ST: strand transfer
AIDS: acquired immunodeficiency syndrome
AUC: area under the pharmacokinetic curve (concentration–time curve) – the change in concentration of the active component in blood plasma or serum over time
СIC_50 _: the inhibitor concentration at which the cytopathogenic effect of the virus in the infected cells decreases by 50%
CIC_95_: the inhibitor concentration at which the cytopathogenic effect of the virus in the infected cells decreases by 95%
* Сl*_p_: clearance, or the extraction ratio – the index showing the rate of extraction of a substance from blood plasma during the biotransformation of this substance, its redistribution in the organism, and excretion
*C*_max _: the maximum or peak of concentration of an active component in blood
EC_50_: inhibitor concentration, at which*in vivo*replication of the virus is suppressed by 50%
EC_90_: inhibitor concentration, at which*in vivo*replication of the virus is suppressed by 90%
*F*: bioavailability – the fraction of a dose of unchanged drug administered orally that reaches the systemic circulation
FBS: fetal bovine serum
FDA: Federal Drug Administration (United States)
IC_50 _: inhibitor concentration, at which the enzyme activity is suppressed by 50%
NHS: normal human serum
PPB: percentage plasma protein binding
*Т*_1/2 _: time by which the concentration of a drug in plasma decreases twice
WT: wild type
